# Three new species of *Herpetogramma* Lederer (Lepidoptera, Crambidae) from China

**DOI:** 10.3897/zookeys.865.35111

**Published:** 2019-07-22

**Authors:** Xiao-Qiang Lu, Ji-Ping Wan, Xi-Cui Du

**Affiliations:** 1 College of Plant Protection, Southwest University, Chongqing, China Southwest University Chongqing China

**Keywords:** DNA barcodes, Maximum Likelihood analysis, morphology, Pyraloidea, Spilomelinae, *
Syllepte invalidalis
*

## Abstract

Five species of the genus *Herpetogramma* in China are studied with morphological and DNA barcode data. *Herpetogrammabiconvexa* Wan, Lu & Du, **sp. nov.**, *H.longispina* Wan, Lu & Du, **sp. nov.**, and *H.brachyacantha* Wan, Lu & Du, **sp. nov.** are described as new. *Herpetogrammarudis* (Warren) and *H.magna* (Butler) are newly diagnosed. Photographs of the habitus and genitalia of these five species are provided.

## Introduction

The genus *Herpetogramma* was established by Lederer (1863) for the type species *H.servalis* Lederer, 1863. There are 103 species of *Herpetogramma* recorded in the Global Information System on Pyraloidea (Nuss et al. 2019). Twenty-two species were recorded in China before our study (Wang 1980; Wang and Speidel 2000; Du 2008; He 2014). This genus was well studied in North America (Solis 2010; Handfield and Handfield 2011; Scholtens and Solis 2015). In regions around China, 10 species were recorded in Korea (Bae et al. 2008; Kim et al. 2012; Roh et al. 2014; Park et al. 2016), 18 in Japan (Yamanaka 1960, 1976; Inoue 1982; Yamanaka 2003; Sasaki and Yamanaka 2013), two in Nepal and three in India (Yamanaka 1995; Mathew 2006). Most species found in these surrounding regions are distributed in China. The adults of most species are brown of various shades with distinct wing patterns, so that light brown, brown, and dark brown are used to describe three general major shades. The genitalia structures are very conservative, exhibiting only subtle differences among species (Shaffer and Munroe 1989; Du 2008, 2009; Solis 2010, 2011; Handfield and Handfield 2011). Dissection is essential to identify species of this genus. In this paper, morphological and DNA barcode data were combined to identify three new species of *Herpetogramma*.

## Materials and methods

### Taxon sampling

Specimens were collected by light trap and killed by ethylacetate or ammonium hydroxide. Genitalia preparation mainly followed the methods introduced by Li and Zheng (1996). Genitalia were examined and described before being mounted on microscope slides. The images of the adults were taken with a digital camera (Nikon P7700) and the images of the genitalia were prepared with a digital camera (Leica DFC 450) attached to a digital microscope (Leica M205 A).

Specimens examined, including types of new species, are deposited in the College of Plant Protection, Southwest University, Chongqing, China (SWUCPP) except for six specimens, including three paratypes, which are deposited in the Insect Collection of the College of Life Science, Nankai University, Tianjin, China (NKU). Among 80 sequences analyzed in the study, 12 sequences were downloaded from the BOLD database at http://v4.boldsystems.org/, two sequences were downloaded from GenBank and 66 newly obtained sequences were deposited in GenBank and can be accessed through the accession numbers listed in Table 1.

**Table 1.** Sample information for the *Herpetogramma* and the outgroup specimens included in the study.

Species	Sequence ID	Location	Accession number
*H.basalis* (Walker, 1866)	SWU201500270	Guangxi, China	MK950840
	SWU201500271	Guangxi, China	MK950841
	SWU201500273	Guangxi, China	MK950842
	SWU201500275	Yunnan, China	MK950843
	SWU201500276	Yunnan, China	MK950844
	–	Madagascar	MIMAD518-15
*H.biconvexa* sp. nov.	SWU201600108	Sichuan, China	MK950798
	SWU201600172	Sichuan, China	MK950792
	SWU201600173	Sichuan, China	MK950793
	SWU201600174	Sichuan, China	MK950793
	SWU201500175	Yunnan, China	MK950794
	SWU201600176	Sichuan, China	MK950796
	SWU201600177	Sichuan, China	MK950797
	SWU201600178	Sichuan, China	MK950790
	SWU201600179	Sichuan, China	MK950791
*H.bipunctalis* (Fabricius, 1794)	SWU201500041	Guangxi, China	MK950820
	SWU201500042	Hainan, China	MK950821
	SWU201500043	Hainan, China	MK950822
*H.brachyacantha* sp. nov.	SWU201600017	Sichuan, China	MK950819
	SWU201600018	Sichuan, China	MK950808
	SWU201600019	Sichuan, China	MK950809
	SWU201600088	Sichuan, China	MK950810
	SWU201600089	Sichuan, China	MK950811
	SWU201600091	Sichuan, China	MK950812
	SWU201600092	Sichuan, China	MK950813
	SWU201600093	Sichuan, China	MK950814
	SWU201600106	Sichuan, China	MK950815
	SWU201600107	Sichuan, China	MK950816
	SWU201600120	Sichuan, China	MK950817
	SWU201500121	Sichuan, China	MK950818
*H.hipponalis* (Walker, 1859)	–	Australia	ANICO104-10
	–	Australia	ANICO105-10
*H.licarsisalis* (Walker, 1859)	SWU201500082	Guangxi, China	MK950830
	SWU201500141	Yunnan, China	MK950828
	SWU201500143	Yunnan, China	MK950829
	SWU201600147	Tibet, China	MK950827
	–	Madagascar	MIMAD522-15
	–	Pakistan	MAMOT958-10
	–	Australia	ANICO091-10
*H.longispina* sp. nov.	SWU201600090	Sichuan, China	MK950799
	SWU201600095	Sichuan, China	MK950800
	SWU201600096	Sichuan, China	MK950801
	SWU201600097	Sichuan, China	MK950802
	SWU201600115	Sichuan, China	MK950803
	SWU201600116	Sichuan, China	MK950804
	SWU201600117	Sichuan, China	MK950805
	SWU201600126	Sichuan, China	MK950806
	SWU201600127	Sichuan, China	MK950807
*H.magna* (Butler, 1879)	SWU201600100	Sichuan, China	MK950823
	SWU201600101	Sichuan, China	MK950824
	SWU201200111	Liaoning, China	MK950825
	SWU201700258	Chongqing, China	MK950826
*H.moderatalis* Christoph, 1881	SWU201600132	Sichuan, China	MK950831
	SWU201500133	Sichuan, China	MK950832
	SWU201200134	Liaoning, China	MK950833
	SWU201500136	Sichuan, China	MK950834
	SWU201200138	Heilongjiang, China	MK950835
	SWU201200139	Jilin, China	MK950836
*H.rudis* (Warren, 1892)	SWU201200003	Chongqing, China	MK950782
	SWU201400006	Guangxi, China	MK950783
	SWU201600007	Shaanxi, China	MK950785
	SWU201600008	Shaanxi, China	MK950786
	SWU201400011	Hubei, China	MK950787
	SWU201600047	Sichuan, China	MK950784
	SWU201700263	Chongqing, China	MK950788
	SWU201700264	Chongqing, China	MK950789
*H.stultalis* (Walker, 1859)	SWU201600243	Tibet, China	MK950837
	SWU201500244	Guizhou, China	MK950838
	SWU201500245	Yunnan, China	MK950839
	–	Papua New Guinea	YAWAN352-14
	–	Papua New Guinea	YAWAN347-14
*H.thestealis* (Walker, 1859)	–	Canada	LBCS753-07
	–	Canada	LBCS752-07
	–	Canada	LBCS404-07
	–	Canada	LBCS402-07
	–	Canada	HM415780
	–	Canada	KT128083
*Syllepteinvalidalis* South, 1901	LXQ1800167	Shanxi, China	MK950779
	LXQ1800168	Hubei, China	MK950780
	LXQ1800234	Chongqing, China	MK950781

### DNA extraction, PCR amplification, and sequencing

In total twelve species of *Herpetogramma* were included for PCR analysis and DNA sequencing (Table 1). Total DNA was extracted from legs of fresh or dry specimens using the TIANGEN DNA extraction kit following the manufacturer’s instructions. The 658-base pair (bp) barcode region of COI was amplified with the LepF1/LepR1 primers (Hajibabaei et al. 2006). PCR products were sent to Sangon Biotechnology Co., Ltd. (Shanghai, China) for sequencing using the aforementioned primers.

### Data analysis

All COI sequences were aligned by MUSCLE 3.8 and corrected by eye after being translated into amino acid sequences (Edgar 2004). Intraspecific and interspecific genetic divergence values were quantified based on the Kimura 2-parameter (K2P) distance model (Kimura 1980) and assessed by MEGA 6.0.6 (Tamura et al. 2013). Phylogenetic analysis was performed based on Maximum Likelihood (ML) with the GTR GAMMA model of nucleotide substitution, and with 1000 bootstrap replicates (Stamatakis et al. 2008). *Syllepteinvalidalis* South, 1901 was chosen as the outgroup as it was never considered to be congeneric with *Herpetogramma*, but is part of the same subfamily (Spilomelinae).

## Results

### DNA sequence analysis

A total of 77 COI sequences of *Herpetogramma* were analyzed. The dataset contained no obvious pseudogenes, supporting the assumption that the correct target gene sequence was amplified and sequenced.

**Figure 1.**
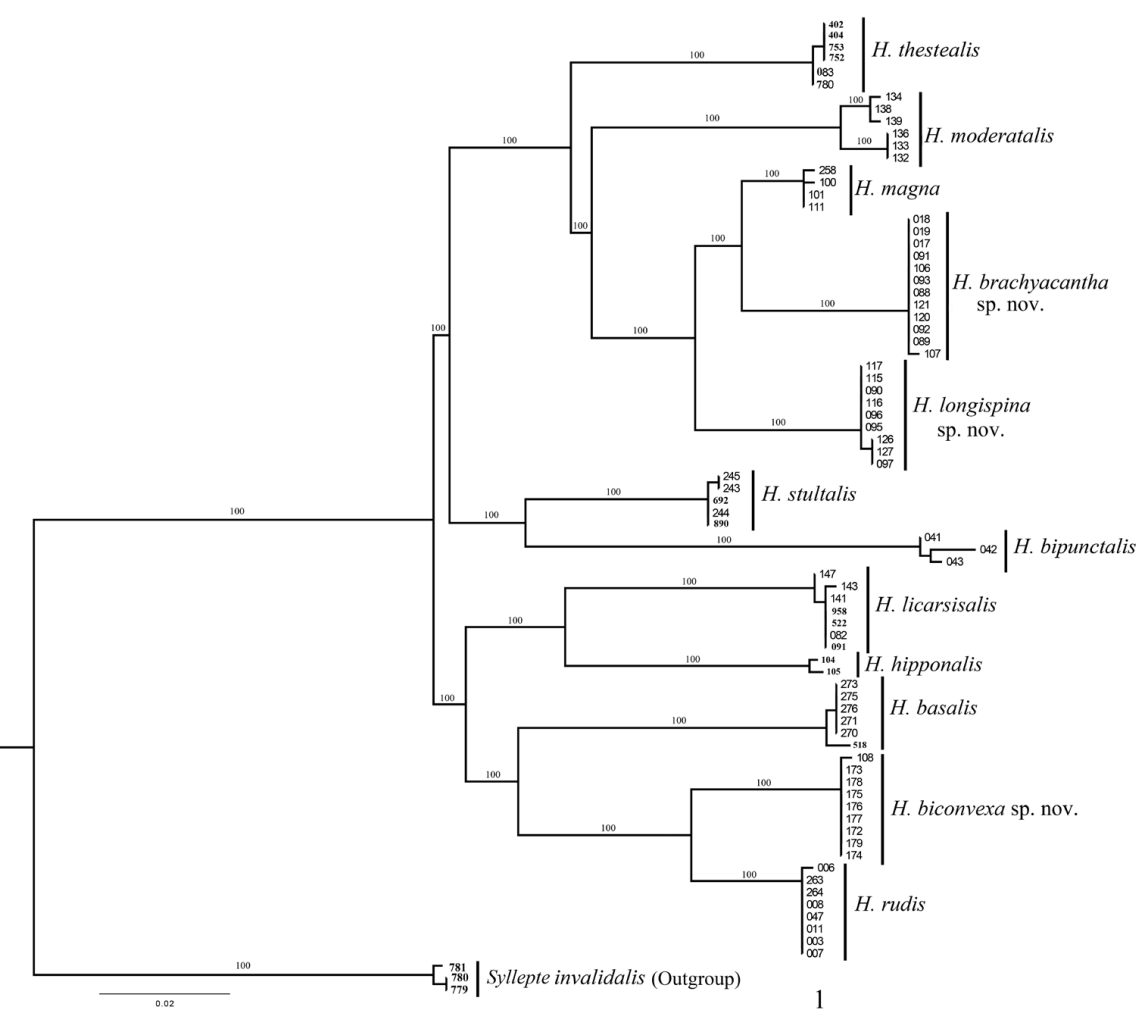
Phylogenetic hypothesis of relationships among 12 species of *Herpetogramma* inferred from a Maximum Likelihood (ML) analysis of the DNA barcode data, with *Syllepteinvalidalis* as outgroup.

We observed 12 monophyletic clades for *Herpetogramma* in the resulting phylogenetic tree (Fig. 1). The pairwise genetic distances within and between these lineages are given in Table 2. Average intraspecific genetic distance ranged from 0.03 to 0.75% while average interspecific genetic distance ranged from 2.93 to 11.17%. The monophyla observed in the phylogenetic analysis were in full congruence with our morphological hypotheses for the investigated species, i.e., our morpho-species hypotheses are in accordance with the barcode clusters recovered (Fig. 1).

**Table 2.** Average Kimura 2-parameter genetic distances in percent, calculated within (in bold) and between species of *Herpetogramma*.

	1	2	3	4	5	6	7	8	9	10	11	12
1. *H.rudis* (*N* = 8)	**0.04**											
2. *H.biconvexa* sp. nov. (*N* = 9)	3.34	**0.03**										
3. *H.basalis* (*N* = 6)	6.96	6.96	**0.15**									
4. *H.longispina* sp. nov. (*N* = 9)	8.31	9.16	8.15	**0.08**								
5. *H.brachyacantha* (*N* = 12)	8.91	9.23	8.08	4.88	**0.03**							
6. *H.magna* (*N* = 4)	8.22	8.35	7.43	3.46	2.93	**0.15**						
7. *H.thestealis* (*N* = 6)	6.39	6.18	6.88	5.78	6.36	5.12	**0.08**					
8. *H.moderatalis* (*N* = 6)	8.98	8.98	8.32	6.64	6.60	6.03	6.93	**0.75**				
9. *H.licarsisalis* (*N* = 7)	7.48	8.40	7.41	9.59	10.02	9.56	8.97	8.58	**0.09**			
10. *H.hipponalis* (*N* = 2)	7.01	8.28	6.93	7.75	8.47	7.95	7.88	7.14	6.29	**0.31**		
11. *H.bipunctalis* (*N* = 3)	8.46	8.53	9.33	9.84	11.17	9.94	8.43	10.41	9.29	9.92	**0.61**	
12. *H.stultalis* (*N* = 5)	7.43	7.94	7.97	8.34	8.54	7.50	7.17	8.01	7.63	8.00	6.98	**0.09**

### Taxonomy

#### Herpetogramma

Taxon classificationAnimaliaLepidopteraCrambidae

Lederer, 1863

b2440224-6431-4a7f-8978-b5dbd0c16edd

Herpetogramma Lederer, 1863: 729. Type species: Herpetogrammaservalis Lederer, 1863, by monotypy.
Pachyzancla Meyrick, 1884: 315. Type species: Botysmutualis Zeller, 1852, by monotypy.
Acharana Moore, [1885]. Type species: Botysotreusalis Walker, 1859, by original designation.
Stenomeles Warren, 1892: 437. Type species: Botysagavealis Walker, 1859, by original designation.
Piloptila Swinhoe, 1894: 142. Type species: Piloptilanigricornalis Swinhoe, 1894, by original designation.
Pantoeocome Warren, 1896: 173. Type species: Pantoeocomedeformis Warren, 1896, by original designation.
Ptiloptila Hampson, 1899: 201. Misspelling.
Stenomelas Hampson, 1912. Misspelling.
Macrobotys Munroe, 1950: 228. Type species: Botysaeglealis Walker, 1859, by original designation.
Coremataria Amsel, 1956: 207. Type species: Botysinfuscalis Guenée, 1854, by original designation and monotypy.
Culcitaria Amsel, 1957: pl. 39, fig. 1. Type species: Botysinfuscalis Guenée, 1854, by monotypy.

##### Diagnosis.

**Figure 2.**
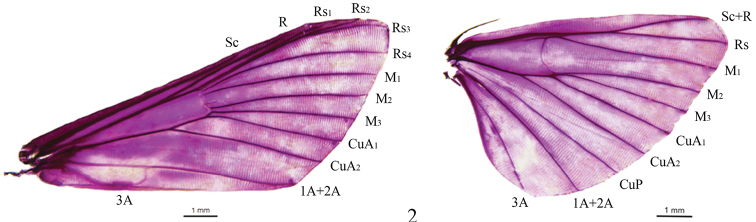
Wing venation of *Herpetogrammarudis* (Warren, 1892).

Frons rounded. Labial palpus obliquely upturned, porrect or upcurved; third segment short and blunt. Maxillary palpi filiform. Male antenna with ventral cilia. Forewing with length of cell ca. half of wing; R from cell at ca. four-fifths above; Rs_1_ closely approximated to Rs_2_+Rs_3_; Rs_4_ curved toward Rs_2_+Rs_3_ at base; M_2_, M_3_ and CuA_1_ from posterior angle of cell, nearly evenly spaced at base; CuA_2_ from cell at four-fifths below. Hindwing with length of cell less than half of wing length; discocellulars arcuate curved; Rs anastomosed with Sc+R ca. basal one-fourth beyond cell; M_2_, M_3_ and CuA_1_ from posterior angle of cell, M_2_ and M_3_ approximated at base; CuA_2_ from cell at two-thirds below (Fig. 2). Male genitalia: Uncus basiconic, narrow normally, with hairs distally; valva at base with small lamellate projection or clasper in some species. Female genitalia: Apophysis anterioris longer than apophysis posterioris; ductus bursae shorter than corpus bursae; corpus bursae with a sub-square signum.

### Checklist of *Herpetogramma* species of China

*H.basalis* (Walker, 1866)

*H.biconvexa* sp. nov.

*H.bipunctalis* (Fabricius, 1794)

*H.brachyacantha* sp. nov.

*H.cynaralis* (Walker, 1859)

*H.dilatatipes* (Walker, 1866)

*H.elongalis* (Warren, 1892)

*H.fuscescens* (Warren, 1892)

*H.hipponalis* (Walker, 1859)

*H.hoozana* (Strand, 1918)

*H.licarsisalis* (Walker, 1859)

*H.longispina* sp. nov.

*H.luctuosalis* (Guenée, 1854)

*H.lulalis* (Strand, 1918)

*H.magna* (Butler, 1879)

*H.mimeticalis* (Hering, 1901)

*H.moderatalis* (Christoph, 1881)

*H.ochrimaculalis* (South, 1901)

*H.okamotoi* Yamanaka, 1976

*H.phaeopteralis* (Guenée, 1854)

*H.pseudomagna* Yamanaka, 1976

*H.rudis* (Warren, 1892)

*H.stultalis* (Walker, 1859)

*H.subalbescens* (Swinhoe, 1894)

*H.submarginalis* (Swinhoe, 1901)

### Key to the new *Herpetogramma* species and their closest relatives based on genitalia

**Table:** 

1	Sacculus with a finger-like projection at basal 2/3 on posterior margin, phallus without cornuti; colliculum adjacent to corpus bursae, signum with a distinct lamellate protuberance along diagonal axis	2
–	Sacculus without finger-like projection on posterior margin, phallus with cornuti; colliculum adjacent to base of ductus bursae, signum slightly depressed along diagonal axis	3
2	Finger-like projection on posterior margin of sacculus with many tiny protrusions	***H.biconvexa* sp. nov.**
–	Finger-like projection on posterior margin of sacculus without protrusions	*** H. rudis ***
3	Phallus with a cluster of long spinose cornuti (at least 1/4 length of phallus)	4
–	Phallus with a cluster of short spinose cornuti (ca. 1/9 length of phallus)	***H.brachyacantha* sp. nov.**
4	Uncus broad, blunt on posterior margin; boundary indistinct between ductus bursae and corpus bursae	***H.longispina* sp. nov.**
–	Uncus narrowed, pointed on posterior margin; boundary distinct between ductus bursae and corpus bursae	*** H. magna ***

#### Herpetogramma biconvexa

Taxon classificationAnimaliaLepidopteraCrambidae

Wan, Lu & Du sp. nov.

275a7e97-0616-4dcc-8b0e-35bf113f800d

http://zoobank.org/2664F46F-0F7C-4DA1-8E39-FBBB1019FE89

Figs 3 , 4 , 13–16

##### Type material.

**Holotype.** ♂, pinned, with genitalia in a separate slide. **China**, **Sichuan**: Pingwu, Wanglang Nature Reserve, Baishagou, 103.55°E, 32.49°N, 3100 m, 20.VII.2016, leg. Ji-Ping Wan, genitalia slide no. WJP17419. **Paratypes. China**, **Sichuan**: 1 ♂, 1 ♀, Pingwu, Wanglang Nature Reserve, Changbaigou, 2900 m, 24.VII.2016, leg. Ji-Ping Wan; 11 ♂♂, 7 ♀♀, Ya’an, Baoxing, Fengtongzhai Nature Reserve, 2180 m, 1–3.VIII.2016, leg. Ji-Ping Wan; 2 ♂♂, Anzihe Nature Reserve, 1312 m, 11 & 15.VII.2016, leg. Ji-Ping Wan; **Yunnan**: 1 ♂, Nanjian, Lingbaoshan Forest Park, 2338 m, 26.VIII.2015, leg. Jing-Xia Zhao & Hao Wei; **Tibet**: 6 ♂♂, 1 ♀, Bomi, Tongmai Town, 2100 m, 21.VII.2016, Jian-Yue Qiu. Genitalia slide no.: WJP16178, WJP17371, WJP17383, WJP17389, WJP17422.

##### Diagnosis.

This species is very similar to *H.rudis* (Warren, 1892), but can be distinguished from the latter by the dark brown wings tinged with pale yellow, forewing length 12.5–15.5 mm (wingspan 29.0–34.0 mm), finger-like projection on the posterior margin of the sacculus broad and with many tiny protrusions, and corpus bursae with central depression ca. half depth of diameter of the corpus burse. In *H.rudis* wings are light brown tinged with white, with a forewing length of 9.0–12.0 mm (a wingspan of 21.0–27.0 mm), the finger-like projection on the posterior margin of the sacculus is slender and without tiny protrusions, and the central depression of the corpus bursae is ca. 1/3 the depth of the diameter of the corpus burse.

##### Description.

**Figures 3–12.**
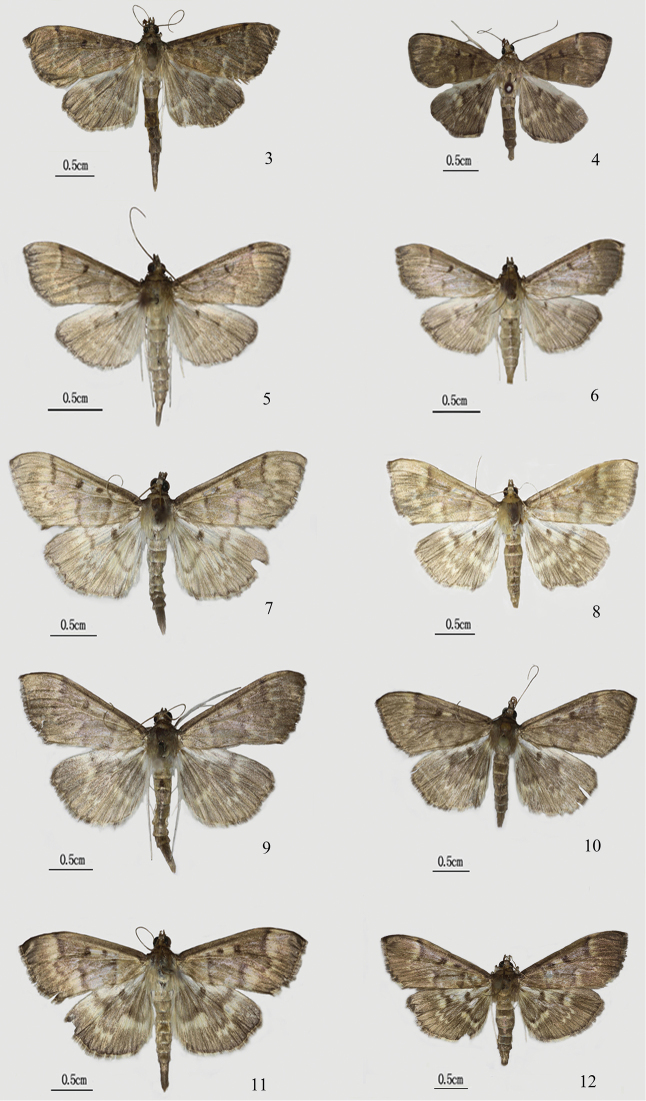
Habitus of *Herpetogramma* species **3, 4***H.biconvexa* sp. nov. **3** male, holotype **4** female, paratype **5, 6***H.rudis***5** male **6** female **7, 8***H.longispina* sp. nov. **7** male, holotype **8** female, paratype **9, 10***H.brachyacantha* sp. nov. **9** male, holotype **10** female, paratype **11, 12***H.magna***11** male **12** female.

Adult (Figs 3, 4): Forewing length 12.5–15.5 mm (wingspan 29.0–34.0 mm). Frons rounded, yellowish brown. Vertex with erect brown scales, white close to eye. Antenna dark brown dorsally, yellowish brown ventrally; male antenna with ventral cilia ca. half as long as diameter of flagellomere. Labial palpus obliquely upturned, basal half white and distal half brown. Thorax and abdomen dark brown dorsally, silvery white ventrally. Legs yellowish white; fore tibia brown distally. Wings dark brown tinged with pale yellow, slightly darker in female. Forewing with orbicular spot and discoidal spot black, the latter reniform; antemedial line dark brown, slightly excurved, adjoined by a light-yellow line inside; postmedial line dark brown, from ca. 2/3 of costa, nearly straight to M_1_, excurved and serrated from M_1_ to CuA_2_, then sharply incurved, and nearly vertical to inner margin below posterior angle of cell, adjoined by a light-yellow line outside. Hindwing with pattern of postmedial line similar to forewing; discoidal spot black. Cilia of wings brown, white along anal angle of hindwing. Abdomen long, second segment with two inconspicuous dark spots basally.

**Figures 13–20.**
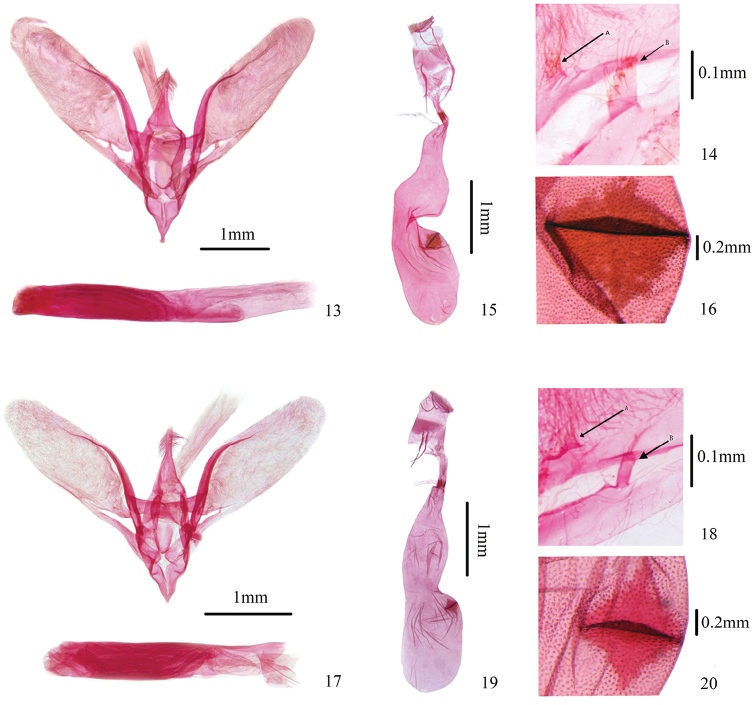
Genitalia of *Herpetogramma* species **13–16***H.biconvexa* sp. nov. **13, 14** male, holotype, genitalia slide no. WJP17419 **15, 16** female, paratype, slide no. WJP17383 **17–20***H.rudis***17, 18** male, genitalia slide no. WJP16318 **19, 20** female, genitalia slide no. WJP16316 **14, 18** A: lamellate projection on base of valva; B: projection on posterior margin of sacculus **16, 20** signum of female genitalia.

**Male genitalia** (Figs 13, 14). Uncus basiconic, distal half narrowed and bearing dorsal hairs, apex pointed and naked. Valva elongate lingulate, densely ciliated, costa slightly widened at middle, and with a small lamellate basal projection bearing hairs distally (Fig. 14A). Sacculus with a broad finger-like projection bearing many tiny protrusions at basal 2/3 of posterior margin, sparse long hairs on top of protrusions (Fig. 14B). Transtilla subtriangular, weakly sclerotized, meeting in middle. Juxta oval, a weakly sclerotized plate. Saccus developed, triangular, distinctly pointed distally. Phallus cylindrical, nearly same length as valva, without cornuti.

**Female genitalia** (Figs 15, 16). Apophysis anterioris ca. twice length of apophysis posterioris. Ductus bursae very short, ca. 1/8 length of corpus bursae; colliculum as long as diameter of ductus bursae, adjacent to corpus bursae. Corpus bursae elongate, elliptical, broad anteriorly, with a central depression ca. half depth of diameter of corpus bursae. Signum square, near depression of corpus bursae, with a distinct lamellate protuberance along diagonal axis (Fig. 16).

**Etymology.** The specific name, *biconvexa*, is derived from the Latin *bi* (meaning ‘two’ or ‘double’) and *convexus* (meaning ‘arched outward’), in reference to the finger-like projection bearing tiny protuberances on the posterior margin of the sacculus.

**Distribution**. China (Sichuan, Yunnan, Tibet).

#### Herpetogramma rudis

Taxon classificationAnimaliaLepidopteraCrambidae

(Warren, 1892)

8675365c-9f28-4a9c-a5b0-17b71e34ea06

Figs 5 , 6 , 17–20

Acharana rudis Warren, 1892: 435.
Psara rudis : Shibuya 1929: 205.
Pachyzancla rudis : Inoue 1955: 182.
Herpetogramma rudis : Lee and Park 1958: 8.
Herpetogramma rude [sic]: Yamanaka and Yoshiyasu 1992: 88.

##### Material examined.

**China**, **Chongqing**: 1 ♂, Simian Mountain Nature Reserve, 1000 m, 20.VII.2012, leg. Xi-Cui Du & Li-Fang; 1 ♂, Simian Mountain Nature Reserve, Wangxiangtai, 900 m, 18.VII.2012, leg. Gui-Qing He; 1 ♂, 1 ♀, Jinyun Mountain Nature Reserve, Shamuyuan, 10–11.IX.2009, leg. Xi-Cui Du; **Yunnan**: 2 ♂♂, Malipo, 1098 m, 4.VI.2015, leg. Man-Fei Tao; 1 ♀, Malipo, Chuantou Town, 193 m, 9.VI.2015, leg. Man-Fei Tao; **Guangxi**: 1 ♂, Jingxi, Tengmao, 672 m, 13.VII.2015, leg. Xu Dan; **Hubei**: 2 ♀♀, Enshi, Xingdou Mountain Nature Reserve, Sanxian, 1200 m, 30.VII.2012, leg. Jun Zhang & Xiao-Bin Fu; **Zhejiang**: 1 ♂, 1 ♀, Tianmu Mountain, Zhonglieci, 400 m, 27.VII.2011, leg. Xi-Cui Du & Xiao-Bin Fu. Genitalia slide no.: WJP16066, WJP16067, WJP16077, WJP16085, WJP16103, WJP16140, WJP16141, WJP16142, WJP16143, WJP16180, WJP 16181, WJP16182, WJP16183, WJP17259, WJP17358, WJP17360, WJP17361.

**Diagnosis.** Adult (Figs 5, 6): Forewing length 9.0–12.0 mm (wingspan 21.0–27.0 mm). Wings light brown tinged with white, lines and spot brown. Forewing with postmedial line excurved and serrated from M_1_ to CuA_2_, adjoined by a light-yellow-white line outside. Male genitalia (Figs 17, 18): Uncus with distal 1/3 narrowed and bearing dorsal hairs, apex pointed and naked. Valva elongate lingulate, with a small basal lamellate projection bearing hairs distally (Fig. 18A). Sacculus with a slender finger-like projection bearing long hairs at basal 2/3 of posterior margin (Fig. 18B). Phallus without cornuti. Female genitalia (Figs 19, 20): Corpus bursae elongate, elliptical, with a central depression ca. 1/3 depth of diameter of corpus bursae. Signum near depression of corpus bursae, with a distinct lamellate protuberance medially along diagonal axis (Fig. 20).

**Distribution**. China (Chongqing, Sichuan, Guizhou, Yunnan, Shaanxi, Henan, Hubei, Anhui, Zhejiang, Fujian, Guangxi, Hainan, Tibet), Korea, Japan, India (Bae et al. 2008; Du 2008, 2009).

**Remarks.** The identification of *H.rudis* was based on the description and photographs of external morphology and genitalia (Warren 1892; Inoue 1982; Bae et al. 2008; Sasaki and Yamanaka 2013). Although species of this genus have similar appearance and conservative genitalia, they can be differentiated according to their subtle and definite characteristics. In addition, it was found that the genitalia of *H.biconvexa* and *H.rudis* were different in size, but the ratios between structures were nearly the same, such as the length ratio between the uncus and the valva at ca. 1:4, ca. 4:3 between the uncus and the saccus, ca. 4:5 between the phallus and the transverse distance of the valva, and nearly 1:7 between the ductus bursae and the corpus bursae. We have not observed these characteristics in other similar species studied.

#### Herpetogramma longispina

Taxon classificationAnimaliaLepidopteraCrambidae

Wan, Lu & Du sp. nov.

ccfd1f85-9ee5-4625-aa4e-9813df17f643

http://zoobank.org/62F95F4C-2374-4699-96EA-46AB0357F7F4

Figs 7 , 8 , 21–25

##### Type material.

**Holotype.** ♂, pinned, with genitalia in a separate slide. **China**, **Sichuan**: Yingjing, Longcanggou, 102°49'22"E, 29°31'5"N, 1610 m, 20.VI.2016, leg. Jian-Yue Qiu, genitalia slide no.: WJP17418. **Paratype. China**, **Sichuan**: 6 ♂♂, 2 ♀♀, Yingjing, Longcanggou, 1610 m, 18–20.VI.2016, leg. Jian-Yue Qiu; 16 ♂♂, 3 ♀♀, Anzihe Nature Reserve, 1312 m, 11–15.VII.2016, leg. Ji-Ping Wan; 1 ♂, 1 ♀, Ya’an, Baoxing, Fengtongzhai, 2180 m, 1.VIII.2016, leg. Ji-Ping Wan; **Hubei**: 1 ♂, 1 ♀, Enshi, Xingdoushan Nature Reserve, Sanxian, 1200 m, 1–2.VIII.2012, leg. Jun Zhang & Xiao-Bin Fu. Genitalia slide no.: WJP17365, WJP17370, WJP17374, WJP17381, WJP17417, WJP17420.

##### Diagnosis.

The species is similar to *H.magna* (Butler, 1879), but can be distinguished from the latter by its light brown wings, the broad uncus blunt at apex and elongate lingulate valva, corpus bursae sharply narrowed posteriorly, and the boundary indistinct between the ductus bursae and corpus bursae. In *H.magna*, wings are brown or dark brown; the narrowed uncus is pointed at apex and the valva is subfusiform, the corpus bursae is slightly narrowed posteriorly, and the boundary is distinct between the ductus bursae and corpus bursae.

##### Description.

Adult (Figs 7, 8). Forewing length 14.5–16.0 mm (wingspan 32.0–34.0 mm). Frons rounded, brown or light brown. Vertex with erect orange-yellow scales. Antenna light brown, male antenna with ventral cilia ca. half as long as diameter of flagellomere. Labial palpus obliquely upturned, basal 2/3 white and distal 1/3 light brown. Thorax and abdomen light brown dorsally, silvery white ventrally. Legs silvery white, fore tibia brown basally. Wings light brown tinged with faint yellow, lines and spots brown, distinct. Forewing with orbicular spot and reniform discoidal spot, faint yellow between orbicular spot and discoidal spot. Antemedial line excurved slightly, adjoined by a light-yellow wider line inside; postmedial line from ca. 2/3 of costa, straight to M_1_, excurved and pointedly serrated from M_1_ to CuA_2_, then sharply incurved, and nearly vertical to inner margin below posterior angle of cell, adjoined by a wide and serrated light-yellow line outside. Hindwing with pattern of postmedial line similar to forewing, discoidal spot reniform. Cilia of wings brown, white along anal angle of hindwing.

**Figures 21–35.**
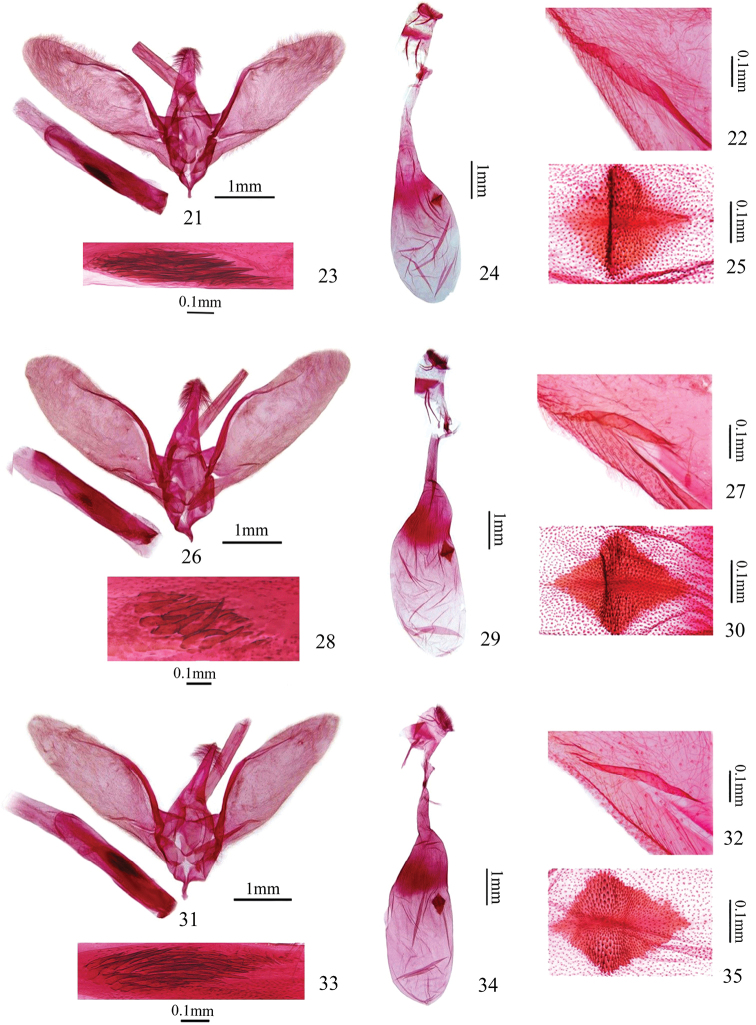
Genitalia of *Herpetogramma* species. **21–25***H.longispina* sp. nov.: **21–23** male, holotype, genitalia slide no. WJP17418 **24, 25** female, paratype, genitalia slide no. WJP17370 **26–30***H.brachyacantha* sp. nov.: **26–28** male, holotype, genitalia slide no. WJP17421 **29, 30** female, paratype, genitalia slide no. WJP17379 **31–35***H.magna*: **31–33** male, genitalia slide no. WJP17423 **34, 35** female, genitalia slide no. WJP17416 **22, 27, 32** lamellate protuberance on base of valva **23, 28, 33** cornuti in male genitalia **25, 30, 35** signum of female genitalia.

**Male genitalia** (Figs 21, 22, 23). Uncus basiconic, broad and shorter, distal 1/3 bearing dorsal setae, apex blunt. Valva elongate lingulate, densely ciliated and bearing a lamellate basal projection (Fig. 22). Juxta cupped, with posterior margin concave and protruding posterolaterally. Saccus subtriangular, short and broad, distinctly pointed distally. Phallus cylindrical; a cluster of long spinose cornuti gathered to subfusiform, ca. 1/4 length of phallus (Fig. 23).

**Female genitalia** (Figs 24, 25). Apophysis anterioris slightly longer than apophysis posterioris. Ductus bursae short, ca. 1/3 length of corpus bursae; colliculum near base of ductus bursae. Boundary indistinct between ductus bursae and corpus bursae. Corpus bursae nearly pear-shaped, sharply narrowed posteriorly, with shallow depression at basal 1/3. Signum nearly square, slightly depressed along diagonal axis (Fig. 25).

##### Etymology.

The specific name, *longispina*, is derived from the Latin *longus* (meaning ‘long’) and *spina* (meaning ‘thorn’), in reference to a cluster of long spinose cornuti in male genitalia.

##### Distribution.

China (Sichuan, Hubei).

#### Herpetogramma brachyacantha

Taxon classificationAnimaliaLepidopteraCrambidae

Wan, Lu & Du sp. nov.

2be29c4d-976b-451a-b806-18e755ca7045

http://zoobank.org/533445AD-7A9C-4FF0-B6A1-2A3EE9C40D6D

Figs 9 , 10 , 26–30

##### Type material.

**Holotype.** ♂, pinned, with genitalia in a separate slide. **China**, **Sichuan**: Anzihe Nature Reserve, 30°N, 1312 m, 15.VII.2016, leg. Ji-Ping Wan, genitalia slide no.: WJP17421. **Paratype. China**: **Sichuan**: 69 ♂♂, 2 ♀♀, Anzihe Nature Reserve, 1312 m, 11–16.VII.2016, leg. Ji-Ping Wan; 1 ♀, Anzihe Nature Reserve, 1690 m, 5.VIII.2015, leg. Xi-Cui Du; 21 ♂♂, 12 ♀♀, Ya’an, Baoxing, Fengtongzhai Nature Reserve, 2180 m, 1–3.VIII.2016, leg. Ji-Ping Wan; 4 ♂♂, Yingjing, Longcanggou, 1610 m, 18–20.VI.2016, leg. Jian-Yue Qiu; 3 ♀♀, Mabian, Yonghong, 1500 m, 23.VII.2004, leg. Ying-Dang Ren (NKU); 1 ♂, Tianquan, Xiaorenyan 1042 m, 9.VII.2012, leg. Jing-Wei Li. Genitalia slide no.: DXC06542, WJP17368, WJP17369, WJP17373, WJP17375, WJP17376, WJP17377, WJP17378, WJP17379, WJP17387, WJP17388, WJP17425.

##### Diagnosis.

The species is similar to *H.longispina* sp. nov. and *H.magna* (Butler). It can be distinguished from them by its wing pattern, which is not as distinct as those of the latter two species. In the male genitalia, its uncus is thinner than that of *H.longispina* and thicker than that of *H.magna*; its cornuti are the shortest, ca. 1/9 of the length of the phallus, while in the latter two species, they are ca. 1/4 of the phallus length. In the female genitalia, the corpus bursae are slightly narrowed posteriorly and the boundary is distinct between ductus bursae and corpus bursae in this species and *H.magna*, while the corpus bursae is sharply narrowed posteriorly and the boundary is indistinct between the ductus bursae and corpus bursae in *H.longispina*.

##### Description

Adult (Figs 9, 10). Forewing length 13.5–15.5 mm (wingspan 31.0–35.0 mm). Frons rounded. Vertex with erect dark brown scales. Antenna brown, male antenna with ventral cilia ca. half as long as diameter of flagellomere. Labial palpus obliquely upturned, basal 2/3 white and distal 1/3 fuscous. Thorax and abdomen dark brown dorsally, silvery white ventrally. Legs silvery white, fore tibia brown distally. Wings brown tinged with faint yellow, lines and spots dark brown and slightly indistinct in female. Forewing with orbicular spot and reniform discoidal spot, faint yellow between orbicular spot and discoidal spot. Antemedial line indistinct, postmedial line from 2/3 of costa, straight to M_1_, excurved and pointedly serrated from M_1_ to CuA_2_, then sharply incurved, and nearly vertical to inner margin below posterior angle of cell, adjoined by a serrated light-yellow line outside. Hindwing with pattern of postmedial line similar to forewing, discoidal spot reniform. Cilia of wings brown, white along anal angle of hindwing.

##### Male genitalia

(Figs 26–28). Uncus basiconic, distal 1/3 bearing dorsal setae, apex slightly blunt. Valva elongate lingulate, densely ciliated and bearing a lamellate basal projection (Fig. 27). Juxta cupped, with posterior margin concave and protruding posterolaterally. Saccus subtriangular, short and broad, pointed distally. Phallus cylindrical; a cluster of short spinose cornuti gathered to subfusiform, ca. 1/9 length of phallus (Fig. 28).

##### Male genitalia

(Figs 29, 30). Apophysis anterioris ca. twice length of apophysis posterioris. Ductus bursae short, ca. 1/3 length of corpus bursae; colliculum near base of ductus bursae. Boundary distinct between ductus bursae and corpus bursae. Corpus bursae nearly pear-shaped, slightly narrowed posteriorly, with shallow depression at basal 1/3. Signum nearly square, near to depression on corpus bursae, slightly depressed along diagonal axis (Fig. 30).

##### Etymology.

The specific name, *brachyacantha*, is derived from the Greek words *brachys* (meaning ‘short’), *ake* (meaning ‘thorn’) and *anthos* (meaning ‘flower’), in reference to a cluster of short spinose cornuti in male genitalia.

##### Distribution.

China (Sichuan).

#### Herpetogramma magna

Taxon classificationAnimaliaLepidopteraCrambidae

(Butler, 1879)

9ed03fbf-eb56-4a79-bd34-246ee9b9becc

Figs 11 , 12 , 31–35

Samea magna Butler, 1879: 74. fig. 2.
Sylepta [sic] magna: Hampson 1898: 723.
Syllepte magna : Inoue 1955: 175.
Herpetogramma magna : Yamanaka 1960: 322.

##### Material examined.

**China**, **Chongqing**: 1 ♂, Chengkou County, Mingzhong Town, 1500 m, 19.VII.2017, leg. Ji-Ping Wan; 2 ♂♂, Chengkou County, Dongan Town, Renhe Village, 1100 m, 28.VI.2013, leg. Gui-Qing He & Li-Jun Xu; 2 ♂♂, Chengkou County, Dongan Town, Xingtian Village, 1300 m, 1.VII.2013, leg. Gui-Qing He & Li-Jun Xu; **Sichuan**: 47 ♂, 7 ♀♀, Nanjiang, Guangwu Mountain, 900 m, 8–9.VII.2013, leg. Gui-Qing He & Li-Jun Xu; 5 ♂♂, 4 ♀♀, Ya’an, Baoxing, Fengtongzhai Nature Reserve, 2180 m, 1–3.VIII.2016, leg. Ji-Ping Wan; 4 ♂♂, Anzihe Nature Reserve, 1312 m, 11–15. VII.2016, leg. Ji-Ping Wan; 2 ♂♂, 1♀, Anzihe Nature Reserve, 1312 m, 4–5.VIII.2015, leg. Xi-Cui Du; 1 ♀, Mabian,Yonghong, 1500 m, 23.VII.2004, leg. Ying-Dang Ren (NKU); 1 ♀, Shimian, Tuanjie Village, 1650 m, 24.VIII.2016, leg. Jian-Yue Qiu & Hao Xu; **Yunnan**: 2 ♂♂, Lijiang, Ninglang, Xichuan Town, 2400 m，31.VII.2013, leg. Gui-Qing He; 1 ♀, Tengchong, Dahaoping Town, 2020 m, 5.VIII.2007, leg. Dan-Dan Zhang; **Liaoning**: 2 ♂♂, 1♀, Huanren, Laotuding, 29.VII.2012, leg. Dan-Dan Zhang & Li-Jun Yang; **Jilin**: 1 ♂, 3 ♀♀, Yanbian, Antu, Wanbao Town, 24.VII.2012, leg. Dan-Dan Zhang; 1 ♂, Linjiang, Huashan Town, Laosandui, 25.VII.2012, leg. Li-Jun Yang; **Hubei**: 1 ♀, Wufeng, Maopin Village, 1175 m, 11.IX.2012, leg. Jin-Wei Li; 2 ♂♂, Xianfeng, Pingbaying, 1280 m, 21.VII.1999, leg. Hou-Hun Li (NKU); 1 ♂, 2 ♀♀, Enshi, Xingdou Mountain, Sanxian, 1200 m, 2.VIII.2012, leg. Jun Zhang; **Shaanxi**: 1 ♀, Yingtou Town, Haopingsi, 1251 m, 17.VII.2012, leg. Jin-Wei Li; 1 ♀, Taibai, Huangbaiyuan Town, 19.VIII.2014, leg. Kai-Li Liu & Jiu-Yang Luo. Genitalia slide no.: DXC06205, DXC06548, WJP17414, WJP17416.

##### Diagnosis.

Adult (Figs 11, 12): Wings brown or dark brown tinged with faint yellow, lines and spots dark brown. Forewing with faint yellow between orbicular spot and discoidal spot, postmedial line excurved and pointedly serrated from M_1_ to CuA_2_, adjoined by a serrated light-yellow line outside. Male genitalia (Figs 31–33): Uncus narrowed, distal 1/3 bearing dorsal setae, apex pointed. Valva subfusiform, densely ciliated and bearing a lamellate basal projection (Fig. 32). Phallus with a cluster of long spinose cornuti gathered to subfusiform, ca. 1/4 the length of the phallus (Fig. 33). Female genitalia (Figs 34, 35): Corpus bursae nearly pear-shaped. Signum nearly square, slightly depressed along diagonal axis (Fig. 35).

##### Distribution.

China (Chongqing, Sichuan, Guizhou, Yunnan, Liaoning, Jilin, Tianjin, Shaanxi, Hubei, Hunan, Taiwan), Korea, Japan, India, Sri Lanka (Hampson 1898; Bae et al. 2008; Du 2008).

##### Remarks.

The identification of this species was based on the description and photographs of external morphology and genitalia (Butler 1879; Inoue 1982; Bae et al. 2008; Sasaki and Yamanaka 2013).

## Discussion

Species of *Herpetogramma* are generally so similar in adult morphology and genitalia that a combined analysis of external morphology, genitalia structures and molecular data is strongly advised for their identification. The three new species described in this paper were discovered by integrating these three sources of data. Over the course of the last decade, an extensive number of specimens of this genus was collected by the members of our laboratory in most regions of China. Judging from preliminary research on these specimens, more new species and/or new records of this genus may be discovered in China in the future. We aim to collect more fresh specimens and obtain more molecular data of *Herpetogramma* species from China and intend to review the whole genus in the future.

## Supplementary Material

XML Treatment for Herpetogramma

XML Treatment for Herpetogramma biconvexa

XML Treatment for Herpetogramma rudis

XML Treatment for Herpetogramma longispina

XML Treatment for Herpetogramma brachyacantha

XML Treatment for Herpetogramma magna

## References

[B1] Amsel HG (1956) Microlepidoptera Venezolana I. Boletin de Entomologia Venezolana, Maracay 10[1954](1–2): 207–208.

[B2] Amsel HG (1957) Microlepidoptera Venezolana II. Boletin de Entomologia Venezolana, Maracay 10[1954](3–4): Berichtigung, 110 pls.

[B3] Bae YS Byun BK Paek MK (2008) Pyralid moths of Korea (Lepidoptera: Pyraloidea). Korea National Arboretum, Samsungad, Seoul, 426 pp.

[B4] Butler AG (1879) Illustrations of typical specimens of LepidopteraHeterocera in the collection of the British Museum. Printed by the order of the trustees, London, 3: 1–82.

[B5] Du XC (2008) Taxonomic Study on Spilomelinae from China (Lepidoptera: Pyraloidea: Crambidae). PhD Thesis, Nankai University, Tianjing.

[B6] Du XC (2009) Spilomelinae In: Li HH, Ren YD et al. (Eds) Insect fauna of Henan (Lepidoptera: Pyraloidea). Science Press, Beijing, 237–305, 359–363, 397–408, 433–440.

[B7] Edgar RC (2004) MUSCLE: multiple sequence alignment with high accuracy and high throughput. Nucleic Acids Research 32: 1792–1797. 10.1093/nar/gkh340 15034147 PMC390337

[B8] Hajibabaei M Janzen DH Burns JM Hallwachs W Hebert PDN (2006) DNA barcodes distinguish species of tropical Lepidoptera. Proceedings of the National Academy of Sciences of the United States of America 4(103): 968–971. 10.1073/pnas.0510466103 PMC1327734 16418261

[B9] Hampson GF (1898) A revision of the moths of the subfamily Pyraustinae and family Pyralidae Part I. Proceedings of the General Meetings for Scientific Business of the Zoological Society of London, 590–761. https://biodiversitylibrary.org/page/30952985

[B10] Hampson GF (1899) A revision of the moths of the subfamily Pyraustinae and family Pyralidae Part II. Proceedings of the General Meetings for Scientific Business of the Zoological Society of London, 172–291. https://biodiversitylibrary.org/page/30951185

[B11] Handfield L Handfield D (2011) A new species of *Herpetogramma* (Lepidoptera, Crambidae, Spilomelinae) from eastern North America. ZooKeys 149: 5–15. 10.3897/zookeys.149.2344 PMC3234405 22207790

[B12] He GQ (2014) Fauna study on Spilomelinae from the Southwest of China (Lepidoptera: Pyraloidea: Crambidae). MSc Thesis, Southwest University, Chongqing.

[B13] Inoue H (1955) Check List of the Lepidoptera of Japan, vol. 2. Rikusuisha, Tokyo, 113−217.

[B14] Inoue H (1982) Pyralidae. In: Inoue H et al. (Eds) Moths of Japan. Kodansha, Tokyo, 307–404 [Vol. 1], 223–254 [Vol. 2].

[B15] Kim SS Bae YS Byun BK (2012) Discovery of two unrecorded species of the family Crambidae (Lepidoptera) from Korea. Korean Journal of Applied Entomology 51: 439–442. 10.5656/KSAE.2012.10.0.056

[B16] Kimura M (1980) A simple method for estimating evolutionary rates of base substitutions through comparative studies of nucleotide sequences. Journal of Molecular Evolution 16: 111–120. 10.1007/BF01731581 7463489

[B17] Lederer J (1863) Beitrag zur Kenntniss der Pyralidinen. Wiener Entomologische Monatschrift 7(8, 10–12): 243–280, 331–504. [pls 2–18]

[B18] Lee JS Park SW (1958) Thirty unrecorded species of Pyralidae from Korea. Korean Journal of Zoology 2: 8.

[B19] Li HH Zheng ZM (1996) Methods and techniques of specimens of Microlepidoptera. Journal of Shaanxi Normal University (natural science edition) 24(3): 63–70.

[B20] Mathew G (2006) An inventory of Indian pyralids (Lepidoptera: Pyralidae). Zoos’ Print Journal, India 21(5): 2245–2258. 10.11609/JoTT.ZPJ.667.2245-58

[B21] Meyrick E (1884) On the classification of the Australian Pyralidina. Transactions of the Entomological Society of London 32: 315.

[B22] Munroe EG (1950) The generic positions of some North American Lepidoptera commonly referred to Pyrausta Schrank (Lepidoptera: Pyralidae). The Canadian Entomologist 82(11): 217–231. 10.4039/Ent82217-11

[B23] Nuss M Landry B Mally R Vegliante F Tränkner A Bauer F Hayden J Segerer A Schouten R H Li Trofimova T Solis MA Prins JD Speidel W (2003–2019) Global Information System on Pyraloidea http://globiz.pyraloidea.org

[B24] Park BS Qi MJ Na SM Lee DJ Kim JW Bae YS (2016) Two newly recorded species of the genus *Herpetogramma* (Lepidoptera: Crambidae: Spilomelinae) in Korea. Journal of Asia-Pacific Biodiversity 9(2): 230–233. 10.1016/j.japb.2016.03.007

[B25] Roh SJ Kim SS Bae YS Byun BK (2014) Four newly recorded species of the family Crambidae (Lepidoptera) from Korea. Animal Systematics, Evolution and Diversity 30(4): 267–273. 10.5635/ASED.2014.30.4.267

[B26] Sammut P (2005) The correct identity of three Pyralidae moths from the Maltese islands (Lepidoptera: Pyralidae). Shilap Revista de Lepidopterologia 33: 235–238.

[B27] Sasaki A Yamanaka H (2013) Spilomelini. In: Nasu Y Hirowatari T Kishida Y (Eds) The Standard of Moths in Japan IV.Gakken Education Publishing, Tokyo, 74–84, 415–478.

[B28] Scholtens BG Solis MA (2015) Annotated check list of the Pyraloidea (Lepidoptera) of America North of Mexico. ZooKeys 535: 1–136. 10.3897/zookeys.535.6086 PMC4669914 26668552

[B29] Shaffer JC Munroe EG (1989) Type material of two African species of *Herpetogramma* and one of *Pleuroptya* (Lepidoptera: Crambidae: Pyraustinae). Proceedings of the Entomological Society of Washington 91: 414–420.

[B30] Solis MA (2010) North American *Herpetogramma* Lederer, 1863 (Lepidoptera: Crambidae: Spilomelinae): type specimens and identity of species in the United States and Canada. Proceedings of the Entomological Society of Washington 112(3): 451–463. 10.4289/0013-8797.112.3.451

[B31] Solis MA (2011) Reassignment of Four Species Currently in *Herpetogramma* Lederer (Spilomelinae) to Pyraustinae (Crambidae: Pyraloidea: Lepidoptera). Proceedings of the Entomological Society of Washington 113(2): 185–194. 10.4289/0013-8797.113.2.185

[B32] Stamatakis A Hoover P Rougemont J (2008) A rapid bootstrap algorithm for the RAxML Web Servers. Systematic Biology 57: 758–771. 10.1080/10635150802429642 18853362

[B33] Swinhoe C (1894) New species of Geometers and Pyrales from the Khasia Hills. Annals and Magazine of Natural History, including Zoology, Botany and Geology, London (ser. 6) 14(80): 142. 10.1080/00222939408677780

[B34] Tamura K Stecher G Peterson D Filipski A Kumar S (2013) MEGA6: Molecular Evolutionary Genetics Analysis version 6.0. Molecular Biology and Evolution 30: 2725–2729. 10.1093/molbev/mst197 24132122 PMC3840312

[B35] Walker F (1859) Pyralides. List of the Specimens of Lepidopterous Insects in the Collection of the British Museum, London 18: 509–798.

[B36] Wang HY Speidel W (2000) Pyraloidea (Pyraloidea, Crambidae). Guide book to insects in Taiwan (19). Shu Shan Books, Taipei, 295 pp.

[B37] Wang PY (1980) China’s Economic Insects (Lepidoptera: Pyralidae). Science Press, Beijing, 206 pp.

[B38] Warren W (1892) Descriptions of new genera and species of Pyralidae contained in the British Museum collection. Annals and Magazine of Natural History, including Zoology, Botany and Geology, London (ser. 6) 9: 429–442. 10.1080/00222939208677357

[B39] Warren W (1896) New genera and species of Pyralidae, Thyrididae, and Epiplemidae Annals and Magazine of Natural History, including Zoology, Botany and Geology, London (ser.6) 17: 173. 10.1080/00222939608680352

[B40] Yamanaka H (1960) On the known and unknown species of the Japanese *Herpetogramma* (Lepidoptera: Pyralididae). Tinea 5(2): 321−327.

[B41] Yamanaka H (1976) Two new species of *Herpetogramma* from Japan, with a note on the known species (Lepidoptera, Pyralidae). Tinea 10(1): 1–6.

[B42] Yamanaka H (1995) Pyralidae of Nepal (I). Tinea 14(Suppl. 2): 182−193.

[B43] Yamanaka H (2003) Descriptions of two new species of the genus *Herpetogramma* Lederer from Japan (Crambidae, Pyraustinae). Tinea 17(5): 225−228.

[B44] Yamanaka H Yoshiyasu Y (1992) Pyralidae In: Heppner JB, Inoue H (Eds) Lepidoptera of Taiwan, 1(2) Checklist. Scientific Publishers, Gainesville, Florida, 77−95.

[B45] Zeller PC (1852) Lepidoptera Microptera, quae J. A. Wahlberg in Caffrorum terra collegit. Kungliga Svenska Vetenskapsakademiens Handlingar, Uppsala and Stockholm 40 (series 3): 1–120.

